# Monitoring and Management of the Palliative Care Patient Symptoms: A Best Practice Implementation Project

**DOI:** 10.3390/nursrep12020035

**Published:** 2022-05-07

**Authors:** Adriana Coelho, Ana Rocha, Daniela Cardoso, Rogério Rodrigues, Cristina Costeira, Sara Gomes, Vitor Parola

**Affiliations:** 1 The Health Sciences Research Unit: Nursing (UICISA:E), Nursing School of Coimbra (ESEnfC), 3004 Coimbra, Portugal; dcardoso@esenfc.pt (D.C.); rogerio@esenfc.pt (R.R.); cristina.costeira@ipleiria.pt (C.C.); vitorparola@esenfc.pt (V.P.); 2 Portugal Centre for Evidence-Based Practice: A JBI Centre of Excellence, 3000 Coimbra, Portugal; 3 Portuguese Oncologic Institute of Coimbra, Palliative Care Unit, 3004 Coimbra, Portugal; 2112@ipocoimbra.min-saude.pt (A.R.); saramrgomes@gmail.com (S.G.); 4 ciTechCare, School of Health Sciences, Polytechnic of Leiria, 2411 Leiria, Portugal

**Keywords:** best practices, implementation science, palliative care, symptom assessment

## Abstract

Background: In palliative care, symptoms are multiple and combined, evolving and changing, with a multidimensional character and multifactorial causes, and a high prevalence, negatively influencing the patient’s and family’s quality of life. Nurses who provide palliative care need to recognize and respond effectively to their patients’ symptoms. Methods: A project will be applied to implement the best practice in monitoring and managing palliative care patients’ symptoms. The Joanna Briggs Institute Practical Application of Clinical Evidence System (JBI PACES) and Getting Research into Practice (GRiP) audit and feedback tool will be used. The JBI PACES and GRiP framework for promoting evidence-based healthcare involves three phases of activity. First, a baseline audit. In a second phase, feedback will be given to the project team after the conclusion of the baseline audit report. Then, a third phase will be conducted as a follow-up audit. Conclusions: This project will improve the practice of the nursing team in monitoring and managing the symptoms of palliative care patients, positively influencing the quality of life of the patient and his family. The implementation and dissemination of this project could boost its replication in other centres.

## 1. Introduction

In palliative care, symptoms are multiple and combined, evolving and changing, with a multidimensional character and multifactorial causes, and a high prevalence, negatively influencing the patient’s quality of life and that of their family [1].

Nurses who provide palliative care need to recognize and respond effectively to their patients’ symptoms. Common symptoms include pain, dyspnea, delirium or terminal restlessness, agitation, and upper airway secretions [2,3,4]. Managing these symptoms can provide comfort and minimize suffering not only of the patient but also their family. Symptom control is one of the essential tools in palliative care [1]. Therefore, nurses need to assess patients so that appropriate interventions may be provided and evaluate the impact of these interventions through regular reassessments [4].

The Best Practice Recommendations mentioned that nurses should consider using an assessment tool identified through the best evidence available to assess and reassess symptoms in patients at end-of-life care (Grade B) [2,3,4]. Indeed, the assessment and recording of symptoms using standardized and validated instruments is a quality indicator of the physical aspects of care that are part of the process indicators in palliative care [5]. One systematic review suggests Edmonton Symptom Assessment System (ESAS), a multidimensional scale, was identified as appropriate for assessing the overall symptoms of patients in the advanced stage of their disease (Level 1) [2].

Additionally, nurses should provide appropriate interventions to manage the symptoms identified during the assessment (Grade A), and nurses providing end-of-life care should receive training to develop their assessment and management skills in this specialized area (Grade A) [4]. Another systematic review suggests the importance of providing education and continuous professional development in this area (Level 1) [6]. Additionally, a pre-post study involving 39 participants shows statistically significant improvements in nurses’ treatment selection and ability to correctly determine patients’ level of dyspnea after a structured training program in this context [7] (Level 2).

This project will be performed in the work institution of one of the coordinators, the Portuguese Oncologic Institute (POI), a specific institute on cancer care located in Portugal. The POI’s mission is to develop actions in healthcare, primary and secondary prevention, research, oncology training and teaching, oncological screening, and oncological registration. Additionally, collaboration in the definition and monitoring of the execution of national cancer policy, constituting itself as a reference institution for the citizens it serves and for health services [7].

The service where we will develop our implementation project will be in the palliative care unit, which has eighteen beds distributed in thirteen nursing rooms. There are twenty-six registered nurses (one in nurse management and twenty-five in clinical practice; six are specialist nurses, fifteen have advanced formation in palliative care, and four have basic formation in palliative care). The group includes twenty-two females and four males, with an average age of forty years. The oldest is fifty-six, and the youngest is twenty-four years old.

The sample will consist of clinical practice nurses (*n* = 25), conscious and oriented inpatients of the palliative care unit of POI (*n* = 20), and one family member of these patients.

This inpatients unit has adults presenting life-threatening oncological illnesses, such as breast, prostate, head and neck, digestive, gynecological, glioblastoma, and other types of advanced cancer.

The importance of efficient and adjusted symptom management is a quality indicator of the nursing care provided. It is crucial to uniformize nursing cares with resources to the best practice available in this area.

In summary, palliative care aims to provide quality of life and comfort [8,9]. For this objective to be achievable, the control of symptoms is crucial. Nevertheless, people hospitalized in palliative care still experience discomfort despite the above [10].

This project aims to improve the practice of the nursing team in the monitoring and management of symptoms of palliative care patients in the POI.

The specific objectives will be:

- To determine current compliance with best practice recommendations for monitoring and management of symptoms in palliative care patients;
- To identify barriers and facilitators to improving compliance;
- To develop strategies to address areas of non-compliance;
- To evaluate changes in compliance with the evidence-based practice recommendations following the implementation of strategies to address identified barriers and enhance identified facilitators in the monitoring and management of symptoms in palliative care patients;
- To improve knowledge regarding best practices regarding symptom monitoring and management in palliative care patients;
- To improve outcomes regarding monitoring and management of symptoms in palliative care patients.

## 2. Materials and Methods

This evidence implementation project will use the JBI PACES and GRiP audit and feedback tools [11]. The JBI PACES and GRiP framework for promoting evidence-based healthcare involves three phases of activity:

(i) Establishing a team for the project and undertaking a baseline audit based on criteria informed by the evidence.
(ii) Reflecting on the baseline audit results and designing and implementing strategies to address non-compliance found in the baseline audit informed by the JBI GRiP framework.
(iii) Conducting a follow-up audit to assess the outcomes of the interventions implemented to improve practice and identify future practice issues to be addressed in subsequent audits.

Ethical approval was given to develop the implementation project (n° 38/2021), despite recognizing that the project will be an active qualitative improvement on activity within the hospital.

This evidence implementation project will be conducted over eight months.

### 2.1. Phase 1: Stakeholder Engagement (or Team Establishment) and Baseline Audit

Phase 1 aims to establish the team for the project and undertake a baseline audit based on criteria informed by the evidence. This phase of the evidence implementation project will be conducted for three months (establishing the team and baseline audit).

The team implementation project will include the coordinators and six registered nurses. Team members, their positions, organizations, and roles will be presented in Table 1.

Table 2 presents the evidence-informed audit criteria that will be used at baseline and follow-up audits, the sample size to be included for each criterion, and the compliance measurement approach (adherence percentage to the criteria stated in the table). The audit criteria (indicators) are based on one JBI Evidence Summaries, “End-of-Life Care: Nursing Assessment” [11,12].

The “audit plan” will be given to all audit nurses.

During the baseline audit period and based on audit criteria 1, the six Registered Nurses of the project team will need to double-check and indicate a “yes” or “no” in the checklist.

To point out a “yes”, first it will be necessary to consult the clinical records on “SClinico” software (software used in Portugal for clinical records) and verify if evidence exists of initial assessment symptoms.

In the second phase, the nurses have to question the patient if someone had asked them characteristics of his symptoms at the moment of their entry into the unit “When you enter in the unit, during the interview realized, anyone asks you characteristics symptoms?” To have a “yes”, two “yes” are needed from the records and the patient or caregiver. Twenty clinical records and 20 patients will be consulted.

For criteria 2, audit nurses need to consult 20 records on the software “SClínico” and check if there exists any evidence recorded on “interventions to manage symptoms”.

These interventions can be pharmacological or non-pharmacological.

To have a “yes,” it is necessary to have a “yes” from the records for pharmacological interventions or non-pharmacological interventions, or “yes” for both.

For criteria 3, audit nurses need to consult 20 records on the software “SClínico” and check if there exists any evidence recorded on “assessment symptoms after intervention”.

Finally, for criterion 4, a questionnaire with two questions will be implemented: “Do you have training in symptom management with a duration equal to or greater than 12 h?” and “Do you have training in the scope of the application of ESAS for a duration equal to or greater than 1 h?”

The possible answers will be “yes” or “no”. To have a “yes” it is necessary to have two “yes” to these questions.

### 2.2. Phase 2: Design and Implementation of Strategies to Improve Practice (GRiP)

In the second phase, all results from the baseline audit will be used to create an audit report and create a webinar with all teams (project team and unit team) to give the first feedback. The second feedback will be made by email.

To understand the barriers that lead to the gap between current practice and the best practice found by the baseline audit. Moreover, in this phase, the team designed strategies to promote the best practices through group meetings. This phase of the evidence implementation project will be conducted for three months.

We will use GRiP software to document barriers, strategies, and resources required and then share all the compiled information with stakeholders. Frequent meetings with the team project to gather opinions and encourage engagement, as programming educational meetings with the palliative care team about monitoring and management of symptoms will be made.

### 2.3. Phase 3: Follow-Up Audit Post Implementation of Change Strategy

The follow-up audit aims to measure if any improvement in compliance with best practice has been reached and recognize any areas that need additional focus and improvement. This phase of the evidence implementation project will be conducted for two months.

The follow-up audit will be performed using the same evidence-based audit criteria as the baseline audit. Additionally, the follow-up audit data will be entered into the PACES software. The baseline audit data will be compared with follow-up audit data to analyze any change in compliance rates.

## 3. Conclusions

The importance of efficient symptom management is an indicator of the quality of nursing care provided. This project will improve the practice of the nursing team in monitoring and managing the symptoms of palliative care patients at the POI, positively influencing the quality of life of the patient and his family. The implementation and dissemination of this project could boost its replication in other centres and even extend it to the entire palliative care team.

## Figures and Tables

**Table 1:** Team members, their positions, organizations, and roles of the implementation project.

Team Member	Position	Organization	Role
Coordinator: Nurse 1 * Nurse 2 **	Registered nurses and Researchers	* Portuguese Oncologic Institute ** Nursing School of Coimbra, Portugal; Health Sciences Research Unit: Nursing; The Portugal Centre for Evidence Based Practice: A Portuguese Oncologic Institute Centre of Excellence	Project coordinator Outlined and monitored clinical Audit project Training Data analysis and Report
Nurse 3	Chief Nurse	study setting	Clinical facilitators
Nurse 4	Training nurse	study setting	Clinical facilitators
Nurse 5	Registered nurse	study setting	Clinical facilitators (champions) Training List barriers Designed strategies Develop some strategies
Nurse 6			
Nurse 7			
Nurse 8			
Nurse 9			
Nurse 10			
Family 1	Family representative	A family member who had a patient hospitalized in the study setting before the implementation project	List barriers Designed strategies

* Corresponds to Nurse 1 affiliation; ** Corresponds to Nurse 2 affiliation.

**Table 2:** Audit criteria, sample, and approach to the measurement of compliance with best practice.

Audit Criterion	Audit Criterion	Sample: (Baseline and Follow-Up Audit)	Method Used to Measure % Compliance with Best Practice
1. Patients are assessed for symptoms using standardized and psychometrically sound assessment tools for end-of-life	Yes: Evidence of record exists AND Patient or caregiver says “yes” No: No evidence of record exists OR The patient says “no” (it is Enough for one “no” to be collected)	*n* = 20 (records)	(1) Records consultation on “SClínico” software (2) Patient (or caregivers) interview
2. Patients receive targeted interventions to manage their symptoms	Yes: Evidence of record exists no: No evidence of record exists	*n* = 20 (records)	Records consultation on “SClínico” software
3. Patients are reassessed using the same standardized and psychometrically sound assessment tools to determine their response to treatment	Yes: Evidence of record exists No: No evidence of record exists	*n* = 20 (records)	Records consultation on “SClínico” software
4. Nurses receive education and continuous professional development related to assessing and managing patients receiving end-of-life care	Yes: nurse answers “yes” to both questions No: nurse answers “no” to one or both questions	*n* = 27 (clinical nurses)	Nurse interview

## Data Availability

Not applicable.
